# Prevention of Elevation in Plasma Triacylglycerol with High-Dose Bezafibrate Treatment Abolishes Insulin Resistance and Attenuates Glucose Intolerance Induced by Short-Term Treatment with Dexamethasone in Rats

**DOI:** 10.1155/2018/3257812

**Published:** 2018-11-08

**Authors:** Maiara Destro Inácio, Alex Rafacho, Nathália Aparecida de Paula Camaforte, Poliana Teixeira, Priscilla Maria Ponce Vareda, Natália Moretti Violato, José Roberto Bosqueiro

**Affiliations:** ^1^Institute of Biosciences, São Paulo State University–UNESP, Botucatu, SP, Brazil; ^2^Department of Physiological Sciences, Center of Biological Sciences, Federal University of Santa Catarina–UFSC, Florianópolis, SC, Brazil; ^3^Department of Physical Education, Faculty of Sciences, São Paulo State University–UNESP, Bauru, SP, Brazil

## Abstract

**Objective:**

Fibrates are used as lipid-lowering drugs and are well tolerated as cotreatments when glucose metabolism disturbances are also present. Synthetic glucocorticoids (GCs) are diabetogenic drugs that cause dyslipidemia, dysglycemia, glucose intolerance, and insulin resistance when in excess. Thus, we aimed to describe the potential of bezafibrate in preventing or attenuating the adverse effects of GCs on glucose and lipid homeostasis.

**Methods:**

Male Wistar rats were treated with high-dose bezafibrate (300 mg/kg, body mass (b.m.)) daily for 28 consecutive days. In the last five days, the rats were also treated with dexamethasone (1 mg/kg, b.m.).

**Results:**

Dexamethasone treatment reduced the body mass gain and food intake, and bezafibrate treatment exerted no impact on these parameters. GC treatment caused an augmentation in fasting and fed glycemia, plasma triacylglycerol and nonesterified fatty acids, and insulinemia, and bezafibrate treatment completely prevented the elevation in plasma triacylglycerol and attenuated all other parameters. Insulin resistance and glucose intolerance induced by GC treatment were abolished and attenuated, respectively, by bezafibrate treatment.

**Conclusion:**

High-dose bezafibrate treatment prevents the increase in plasma triacylglycerol and the development of insulin resistance and attenuates glucose intolerance in rats caused by GC treatment, indicating the involvement of dyslipidemia in the GC-induced insulin resistance.

## 1. Introduction

Glucose and lipid metabolism is a process continuously regulated by several humoral and neural factors that guarantee the constant physiological supply of circulating energetic substrates (i.e., glucose and free fatty acids). Disruption of such a fine process can lead to dyslipidemia or dysglycemia, which are both risk factors for cardiovascular and metabolic diseases (i.e., hypertension and diabetes mellitus) [1]. The worldwide increasing prevalence of metabolic-related diseases, including obesity, hypertension, and diabetes mellitus type 2 (DM2), is associated with numerous factors, such as genetic background, epigenetics (i.e., intrauterine influences), and lifestyle (i.e., physical inactivity and hypercaloric diets) as well as indirect ingestion of endocrine disruptor compounds (i.e., pesticides and bisphenol A) [1].

In addition to these well-known risk factors, exposure to prolonged treatment or high dosages of glucocorticoids (GCs) may also be a concern of glucose and lipid metabolism [2]. GC-based treatments are typically associated with glucose and lipid disturbances (i.e., glucose intolerance, reduction in insulin sensitivity, dyslipidemias of varied degrees, and hyperinsulinemia), which are well reproducible in both rodents [3–5] and in humans [6, 7]. GCs exert such an effect upon the reduction of glucose disposal and increased plasma triacylglycerol levels, which typically result from the attenuation of insulin-stimulated glucose uptake in skeletal muscles, attenuation of insulin suppression on liver glucose output, and attenuation of insulin-suppressive action on adipose tissue lipolysis [2]. These low responses to insulin in peripheral tissues, also termed as peripheral insulin resistance, may be related to increased plasma lipid abundance, which ultimately can be accumulated ectopically in nonadipose tissues [8].

A few preclinical [9] and clinical studies [10] have demonstrated the positive impact of glucose-lowering drugs (i.e., metformin, pioglitazone) coadministered together or prior to treatment with GCs. However, none of these glucose-lowering drugs were developed to act primarily on lipid metabolism. Considering that the elevation of plasma free fatty acids may be involved with the genesis of insulin resistance [8], the coadministration of lipid-lowering drugs with GCs could offer insight. In this sense, bezafibrate is an ideal candidate. Fibrates are a class of drugs with better results for the management of elevation in plasma triacylglycerol than statins (statins are primarily indicated to reduce LDL cholesterol levels), especially when glucose metabolism is also impaired [11].

Considering that dexamethasone treatment induces marked hypertriacylglycerolemia together with reduced insulin sensitivity, glucose intolerance, and hyperinsulinemia in adult rats [3–5], we explored the potential of bezafibrate treatment in preventing the elevation of plasma triacylglycerol and other adverse metabolic actions induced by dexamethasone treatment. In view of GCs in excess induces robust genomic-mediated adverse effects [2] that may surpass the possible therapeutic actions of fibrates (if it is introduced after GC), our study design resides in a hypothetical context where fibrates are being used prior to GCs (e.g., a patient that is on continuous bezafibrate treatment and needs to be exposed to a therapy based on GC action). Furthermore, we expect that with this design, the GC-induced elevation in plasma triacylglycerol will be minimal. We hypothesized that previous treatment with bezafibrate will attenuate the elevation in triacylglycerol levels caused by GC treatment and protect rats from the GC-induced side effects on glucose metabolism. Our main results demonstrate that the introduction of high-dose bezafibrate treatment prior to GC treatment prevented the increase in plasma triacylglycerol and advancement of insulin resistance and attenuated the glucose intolerance in rats, indicating the involvement of dyslipidemia in GC-induced insulin resistance.

## 2. Materials and Methods

### 2.1. Ethical Approval

The experiments with rats were approved by the Institutional São Paulo State University Committee for Ethics in Animal Experimentation (approval ID: 2150/46/01/09) in accordance with the National Council for Animal Experimentation Control (CONCEA).

### 2.2. Materials

Dexamethasone phosphate (Decadron^®^) was purchased from Aché (Campinas, SP, Brazil), and Bezafibrate was purchased from Pharma Nostra (Campinas, SP, Brazil). Gum Arabic was obtained from Sigma (St. Louis, MO, USA). Regular human recombinant insulin (Biohulin^®^) was acquired from Biobrás (Montes Claros, MG, Brazil). Sodium thiopental (Thiopentax) was purchased from Cristália (Itapira, SP, Brazil). The ^125^I-labelled insulin (human recombinant) for RIA assay was purchased from Amersham Biosciences (Little Chalfont, Buckinghamshire, UK). The reagents were used in the glucose tolerance test, and the hepatic glycogen, insulin secretion, and RIA protocols were acquired from LabSynth (Diadema, SP, Brazil) and Sigma. The enzymatic colorimetric assay for the quantification of nonesterified fatty acid (NEFA) was obtained from Wako Chemicals USA Inc. (Richmond, VA, USA). The enzymatic colorimetric assays for the quantification of triacylglycerol and total cholesterol (T-Chol) from detection kits were purchased from InVitro (Itabira, SP, Brazil). Plasma insulin was quantified by RIA using a gamma alpha counter (Perkin Elmer, Waltham, MA, USA) as previously described [4, 5].

### 2.3. Animals

Experiments were performed on 4 groups of 40 male Wistar rats (defined as sets 1, 2, 3, and 4), accounting for a total of 160 rats. The rats were obtained from the UNESP Animal Care Unit, Campus of Botucatu, and maintained at 22 ± 2°C on a 12 h light-dark cycle (lights on at 0600, lights off at 1800). The rats had access to food (commercial standard chow for rats, BIOBASE^®^ 9301; Águas Frias, SC, Brazil) and water *ad libitum*.

### 2.4. Bezafibrate and Dexamethasone Treatments

**Figure 1:**
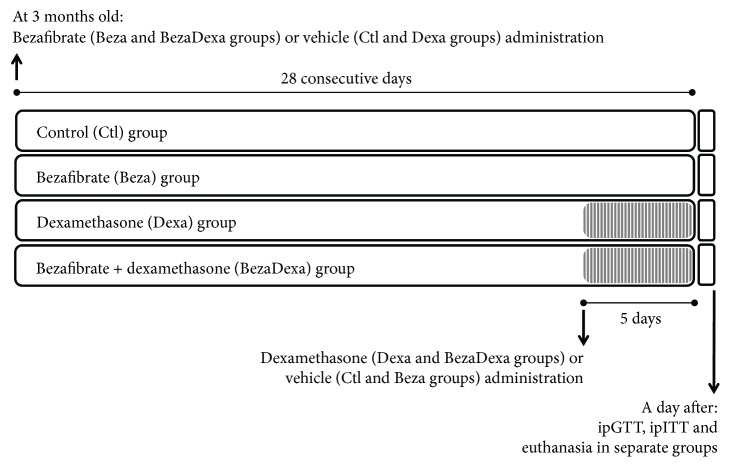
Experimental design. At 3 months old, rats ingested bezafibrate ((300 mg/kg of body mass (b.m.)) once a day via oral gastric gavage administration between 0700 and 0800 h, diluted in 5% gum Arabic, for 28 consecutive days, while control group received a daily gavage administration of vehicle alone (equivalent to 1 ml/kg of b.m.). In the last 5 days of bezafibrate treatment, half of rats under bezafibrate treatment received daily intraperitoneal (i.p.) injection of dexamethasone phosphate (equivalent to 1 mg/kg of dexamethasone, b.m., diluted in saline solution), between 0700 and 0800 h, while half of rats from the control group received daily i.p. injection of vehicle (0.9% NaCl, at 1 ml/kg of b.m.). A day after the last dexamethasone or vehicle administration, separate groups of rats were submitted to ipGTT, ipITT, and euthanasia for biochemical data analyses and ex vivo experiments. ipGTT: intraperitoneal glucose tolerance test; ipITT: intraperitoneal insulin tolerance test.

According to Figure 1, rats aged 28 days were housed for acclimatization until they reached 3 months old and subsequently separated in four groups: bezafibrate group (Beza) received a daily oral gastric (gavage) administration of bezafibrate ((300 mg/kg of body mass (b.m.)), between 0700 and 0800 h, diluted in 5% gum Arabic, for 28 consecutive days; control group (Ctl) received a daily gavage administration of vehicle alone (5% gum Arabic), between 0700 and 0800 h, equivalent to 1 ml/kg of b.m., for 28 consecutive days; bezafibrate plus dexamethasone group (BezaDexa), in the last 5 days of bezafibrate treatment, half of rats from the Beza group received daily intraperitoneal (i.p.) injection of dexamethasone phosphate (equivalent to 1 mg/kg of dexamethasone, b.m., diluted in saline solution), between 0700 and 0800 h; dexamethasone group (Dexa), in the last 5 days of vehicle treatment, half of rats from the Ctl group received daily i.p. injection of vehicle (0.9% NaCl, at 1 ml/kg of b.m.), between 0700 and 0800 h. The administration of bezafibrate reduces plasma triacylglycerol in normal rats [12] and hyperglycemic rat models (OLETF rats) [13, 14]. Although the dose of bezafibrate used in the present study was 2 to 3 times higher than that used in previous studies, this dose was still 25% lower than the dose where only mild and transitional hepatic toxicity was observed in rats [15]. We opted for this high dose since there is evidence that low dosages have no persistent effect on reducing plasma triacylglycerol and NEFA values in a rat model of dyslipidemia [14]. With this high bezafibrate dose, we generated a rat model where the influence of circulating triacylglycerol was not present among the outcomes produced by dexamethasone treatment. This pharmacological intervention permitted us to observe how much plasma triacylglycerol could be involved in the dexamethasone adverse effects on glucose homeostasis. Dexamethasone, at the dose and period administered, produces significant adverse effects on glucose metabolism, typical of prediabetes, including glucose intolerance, reduction of insulin sensitivity, augmented fasting blood glycemia and plasma triacylglycerol, and increased pancreatic beta cell mass, which makes this compound a valuable tool for challenging glucose metabolism experimentally [16, 17].

### 2.5. Metabolic Measurements (Set 1)

Body mass was measured daily from the beginning of bezafibrate treatment until the day of euthanasia using a conventional electronic balance (Tecnal, Piracicaba, Brazil). The growth rate was determined based on the formula [((final b.m–initial b.m.)/(initial b.m.)) × 100]. Food intake was measured only during the period of dexamethasone administration to verify the reproducibility of its anorexigenic action [5] and how bezafibrate impacts on it. For this, the remaining chow after a 24 h period was normalized to the total body mass from each cage as previously described [5, 17]. On the day after the last day of dexamethasone treatment, separate groups of fasted (12–14 h) and fed rats had blood collected from the tail to measure blood glucose levels with a glucometer (One Touch, Johnson & Johnson, NJ, USA). Immediately after blood collection, the rats were euthanized (exposure to CO_2_ followed by decapitation), and the trunk blood was collected in EDTA-NaF-containing tubes (Glistab-Labtest; Lagoa Santa, MG, Brazil) to obtain the plasma. The plasma, obtained after blood centrifugation (600 *×*g), was stored at −80°C in several aliquots for posterior measurement of insulinemia, plasma triacylglycerol, total cholesterol, and circulating NEFA with commercial kits according to the manufacturer's instructions. The organs (listed in Table 1) were gently withdrawn and weighed.

### 2.6. Liver Glycogen (Set 1)

Determination of hepatic glycogen content was performed as previously described [4, 5, 16]. Glycogen was determined by a phenol-based assay using a spectrophotometer.

### 2.7. Intraperitoneal Glucose Tolerance Test (ipGTT) and In Vivo Glucose-Stimulated Insulin Secretion (Set 2)

On the day after the last day of treatment of each group, fasted (12–14 h) rats were used for the ipGTT experiments. The tail tip of anesthetized rats (60 mg/kg Thiopentax, b.m., i.p.) was cut for blood collection after assessing the absence of reflexes. The first drop was discarded, and the second drop was used for the determination of glycemia (time 0) using a glucometer as previously described. A 50% glucose solution prewarmed at 36°C (2 g/kg, b.m., i.p.) was immediately administered, and blood samples were collected from the tail tip at 15, 30, 60, 90, and 120 min for blood glucose measurements [16, 17]. Area under curve (AUC) was obtained after normalization by the initial blood glucose value. At 0, 15, and 120 minutes, additional blood volumes (80 *μ*l) were collected in anticoagulant-containing tubes for plasma separation and the posterior quantification of insulin by RIA as a measure of the *in vivo* glucose-stimulated insulin secretion [4, 16].

### 2.8. Intraperitoneal Insulin Tolerance Test (ipITT) (Set 3)

On the day after the last day of treatment in each group, fed rats were used for ipITT experiments. The rats were anesthetized as for ipGTT and tail tips were cut for blood collection. The first drop of blood was discarded, and the second drop was used for the determination of glycemia (time 0) using a glucometer. Immediately, human recombinant insulin prewarmed at 36°C (equivalent to 2 IU/kg b.m., i.p.) was administered. Additional samples were collected at 10, 20, and 30 min for blood glucose measurement. The constant rate for glucose disappearance (*K*_ITT_) was calculated from the slope of the regression line obtained with log-transformed glucose values between 0 and 30 min after insulin administration (linear phase of glucose decay) [4, 16, 17].

### 2.9. Isolation of Islet and Glucose-Stimulated Insulin Secretion (Set 4)

The islets were isolated by collagenase digestion of the pancreas as previously described [4, 5, 16, 17]. For static incubation, groups of five islets were first incubated for 1 h at 37°C in 1 ml Krebs bicarbonate buffer solution of the following composition (in mM): 115 NaCl, 5 KCl, 2.56 CaCl2, 1 MgCl2, 24 NaHCO3, 15 HEPES, and 5.6 glucose, supplemented with 0.1% of bovine serum albumin and equilibrated with a mixture of 95% O_2_ : 5% CO_2_, pH 7.4. The medium was subsequently replaced with fresh buffer containing 2.8 or 16.7 mM glucose and further incubated for 1 h. At the end of the incubation, the samples were stored at −20°C for subsequent measurement of the insulin content by RIA. Total islet insulin content was determined in separate pools of islets (four different pools consisting of 50 islets each) after extraction in acid ethanol solution (12 mM HCl in 70% ethanol). The average insulin content, obtained from these pools, was used for estimation of insulin secretion in each group based on total islet insulin content as previously described [4]. All islets handpicked for insulin secretion experiments were selected based in their spherical uniformity, integrity, and size to avoid any bias of islet aspect and size.

### 2.10. Statistical Analysis

The results are expressed as the mean ± SEM (points and connecting line) and the mean ± SD or the median and interquartile range (scatter plot with bar) of the indicated number (*n*) of animals. The symmetry of the data was tested by Kolmogorov-Smirnov and Shapiro-Wilk's normality tests. Analysis of variance (ANOVA) (two-way ANOVA) for unpaired groups, followed by Tukey's post hoc test, was utilized for multiple comparisons. The extreme studentized deviate method was applied to determine whether any of the values was a significant outlier (Grubb's test from online available GraphPad QuickCalcs). References in the text to “their respective control groups” means differences among Dexa and BezaDexa groups vs. Ctl and Beza groups, respectively (effect of dexamethasone), or Beza and BezaDexa groups vs. Ctl and Dexa groups, respectively (effect of bezafibrate). Significance was set at *p* < 0.05.

## 3. Results

### 3.1. Bezafibrate Treatment Had a Slight Impact on the Anorexigenic Effect of Dexamethasone Treatment

**Figure 2:**
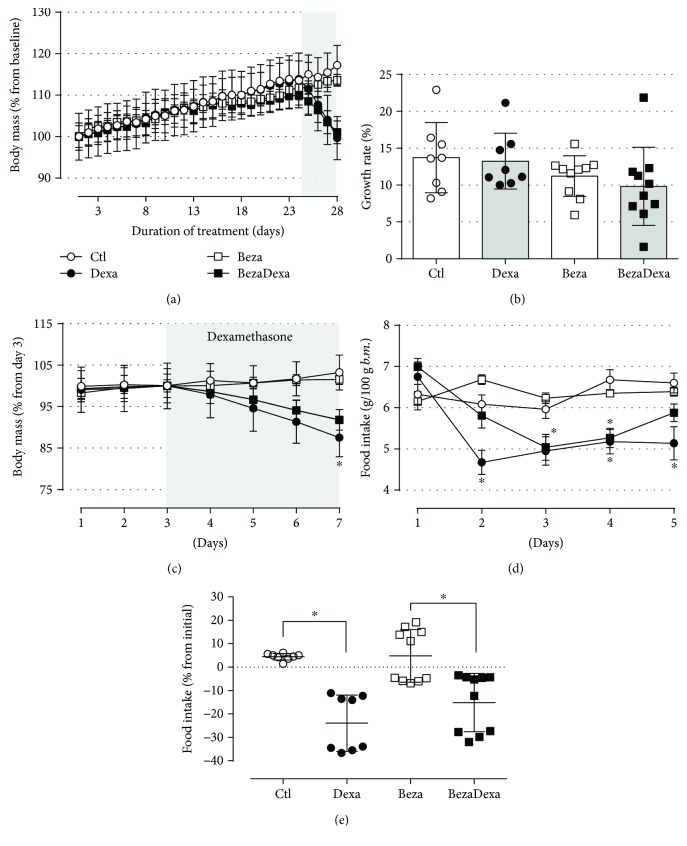
Body mass and relative food intake. (a) The average body mass values during the treatments with bezafibrate and dexamethasone. (b) The estimated growth rate (based on 1st and 23rd days) where no differences were observed between the groups. (c) The average body mass values during the treatment with dexamethasone. Observe the reduction in body mass by an effect of dexamethasone treatment. (d) Relative food intake during glucocorticoid treatment and (e) the percentage of reduction in the food intake from the end to the initial treatment with glucocorticoid. We can observe a reduction of relative food intake by an effect of dexamethasone. Results are expressed as mean ± SEM in (a, c, and d) and mean ± SD in (b) and (e). In (a, c, and d), the variances were expressed as standard error of the mean (SEM) for esthetic reasons. ^∗^Significantly different vs. the respective control group (dexamethasone effect) using two-way ANOVA with Tukey's post hoc test. *n* = 8–10, *p* < 0.05.

Before initiating bezafibrate treatment, the rats in all groups exhibited similar body masses, and bezafibrate administration for 28 consecutive days changed neither the body mass (Figure 2(a)) nor the estimated growth rate (based on 1 and 23 days) (Figure 2(b)). Dexamethasone treatment resulted in a reduced final body mass (−15%) in the Dexa group compared with that in the Ctl group (Figure 2(c)) (*n* = 8–10, *p* < 0.05), but the 10% reduction was not significant in the BezaDexa group compared with that in the Beza group. Relative food intake was similar among the four groups prior to the initiation of dexamethasone treatment (morning of the first dexamethasone injection) but was significantly lower in the Dexa and BezaDexa groups vs. their respective controls, after 1 and 2 days of dexamethasone treatment, respectively (Figure 2(d)) (*n* = 8–10, *p* < 0.05), which remained consistent until the end of treatment in the Dexa group. The estimation of food intake between the final and initial dexamethasone treatment revealed no negative or positive impact of bezafibrate administration on the anorexigenic effect of GC (Figure 2(d)). Altogether, treatment with bezafibrate before and during the administration dexamethasone had no major impact on the anorexigenic action of high doses of GC treatment.

### 3.2. Bezafibrate Treatment Abolished the GC-Induced Elevation in Plasma Triacylglycerol and Improved Basal Glycemic Values

**Figure 3:**
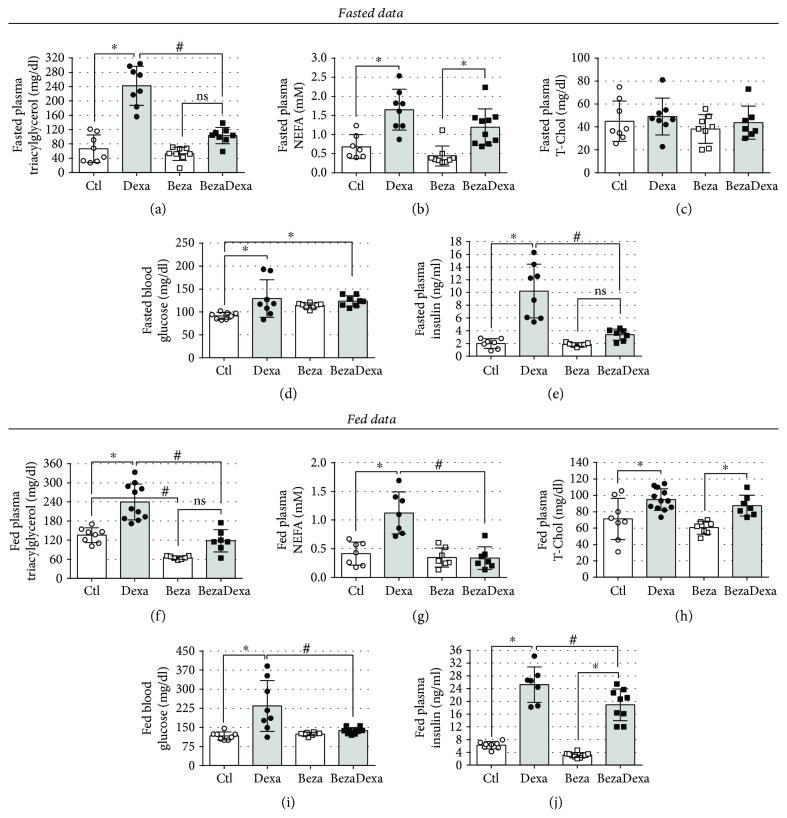
Biochemical data. Fasting plasma (a) triacylglycerol, (b) NEFA, (c) T-Chol, (d) blood glucose, and (e) plasma insulin values. Observe the marked effect of bezafibrate treatment on prevention of elevation of triacylglycerolemia and insulinemia caused by dexamethasone treatment. Fed plasma (f) triacylglycerol, (g) NEFA, (h) T-Chol, (i) blood glucose, and (j) plasma insulin values. We can note the marked effect of bezafibrate treatment on prevention of elevation of plasma triacylglycerol and NEFA and blood glucose. Results are expressed as mean ± SD. ^∗^Significantly different vs. the respective control group (dexamethasone effect) and ^#^significantly different vs. the respective control group (bezafibrate effect) using two-way ANOVA with Tukey's post hoc test. *n* = 7–12, *p* < 0.05. NEFA: nonesterified fatty acid; T-Chol: total cholesterol; ns: not significant.

To verify the efficacy of bezafibrate treatment, we quantified circulating triacylglycerol, NEFA, and T-Chol both at fasted and fed states. In fact, bezafibrate administration prevented any increase in the plasma triacylglycerol caused by GC treatment in both metabolic states (Figures 3(a) and 3(f)) (*n* = 7–11, *p* < 0.05). Bezafibrate treatment exerted no significant impact on the elevated fasting NEFA values but prevented the increase in fasting blood glucose as well as plasma insulin caused by dexamethasone administration (Figures 3(b)–3(e)) (*n* = 7–10, *p* < 0.05). In the fed state, bezafibrate treatment had no major impact on the elevated plasma T-Chol values but abolished the rise in NEFA and blood glucose values and attenuated the elevation in plasma insulin values induced by GC treatment (Figures 3(g)–3(j)) (*n* = 7–12, *p* < 0.05). Bezafibrate had no negative impact per se on any of the parameters evaluated when compared with the Ctl group (Figures 3(a)–3(j)). Evaluation of relative visceral fat mass revealed no impact of dexamethasone nor from bezafibrate treatment (Table 1) (*n* = 7–8). Dexamethasone-treated rats exhibited lower relative spleen masses and elevation in the hepatic glycogen content, as a result of GC treatment, which was completely abolished (glycogen data) in rats from the BezaDexa group (Table 1) (*n* = 7–8, *p* < 0.05). Thus, the prevention of the elevation in triacylglycerol levels with bezafibrate treatment was associated with improvements of GC-induced glucose and lipid metabolism dysfunctions.

### 3.3. Bezafibrate Treatment Improved the Glucose Intolerance and Prevented the Insulin Resistance Caused by High-Dose GC Treatment

**Figure 4:**
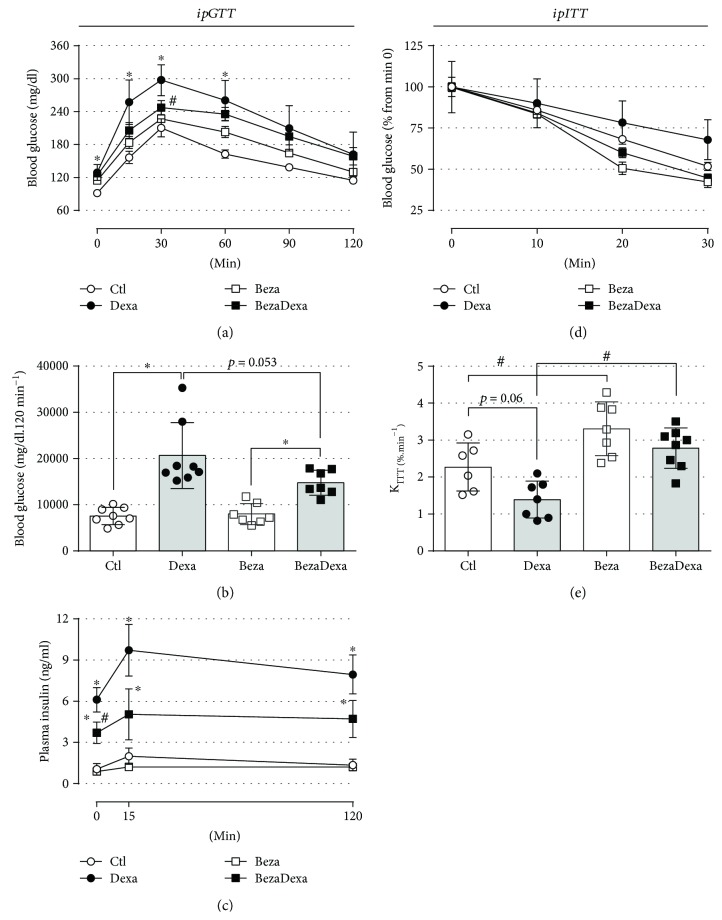
Glucose tolerance and insulin sensitivity. (a) The average blood glucose values during an intraperitoneal glucose tolerance test (ipGTT) (2 g/kg, body mass), (b) the average values of area under curve (AUC), and (c) the plasma insulin in response to glucose load. Bezafibrate treatment attenuated the glucose intolerance caused by the dexamethasone treatment in the BezaDexa group. (d) The average of normalized blood glucose values (as % from min 0) during an intraperitoneal insulin tolerance test (ipITT) (2 IU/kg, body mass) and (e) the respective constant for glucose disappearance (*K*_ITT_). Bezafibrate treatment prevented the insulin resistance caused by the dexamethasone treatment in the BezaDexa group. Results are expressed as mean ± SEM in (a, c, and d) and as mean ± SD in (b) and (e). In (a, c, and d), the variances were expressed as standard error of the mean (SEM) for esthetic reasons. ^∗^Significantly different vs. the respective control group (dexamethasone effect) and ^#^significantly different vs. the respective control group (bezafibrate effect) using two-way ANOVA with Tukey's post hoc test. *n* = 6–8, *p* < 0.05.

Considering that glucose intolerance and reduction in insulin sensitivity commonly occur together with basal dysglycemia and dyslipidemia, we assessed these two aspects in rats treated with bezafibrate to verify the impact of such treatment on these parameters. During the first 60 min of the glucose tolerance test, only the Dexa group presented higher levels of blood glucose in relation to the Ctl group (Figure 4(a)) (*n* = 7–8, *p* < 0.05). Blood glucose values at min 30 were 210 ± 16, 297 ± 28, 227 ± 18, and 247 ± 12 mg/dl for the Ctl, Dexa, Beza, and BezaDexa groups, respectively. Treatment with bezafibrate had no effect per se (in relation to the Ctl group) but attenuated the effect of dexamethasone treatment on glucose tolerance as observed during GTT experiment (Figure 4(a)) (30 min for the BezaDexa group) and by the AUC values (Figure 4(b)) (*n* = 7-8). This positive impact of bezafibrate treatment upon glucose tolerance in rats treated with GC was associated with enhanced beta cell function (see insulin secretion in response to glucose along the GTT experiment in the BezaDexa group related to the Beza group) (Figure 4(c), *n* = 6–8) and the complete prevention of peripheral insulin resistance, as demonstrated in the insulin tolerance test (Figure 4(d), *n* = 6–8) and the estimation for the constant rate for glucose disappearance (*K*_ITT_) (Figure 4(e), *n* = 6–8). Bezafibrate treatment for 28 days had an effect per se, leading to an enhanced insulin sensitivity compared with the Ctl group. These data suggested that pharmacological prevention of elevation in plasma triacylglycerol levels was associated with improved glucose tolerance and insulin sensitivity during exposure to a high GC dose.

### 3.4. Islets from Rats Treated with Bezafibrate Were More Responsive to High Glucose

**Figure 5:**
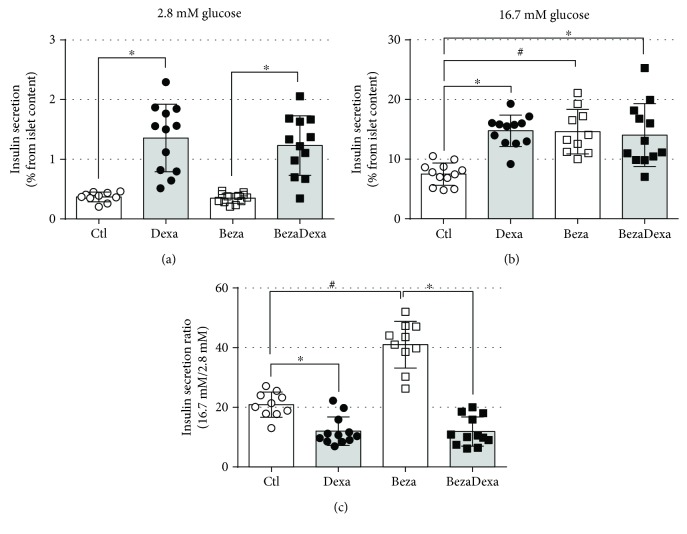
Glucose-stimulated insulin secretion. Insulin response to (a) 2.8 mM and (b) 16.7 mM of glucose in freshly isolated islets. (c) Insulin secretion ratio between 16.7 mM and 2.8 mM glucose. Rats from Beza group also had higher insulin response to high glucose concentration. Results are expressed as mean ± SD. ^∗^Significantly different vs. the respective control group (dexamethasone effect) and ^#^significantly different vs. the respective control group (bezafibrate effect) using two-way ANOVA with Tukey's post hoc test. *n* = 10–12 wells from 2 independent experiments, *p* < 0.05.

To verify whether bezafibrate treatment influenced beta cell function, we evaluated the glucose-stimulated insulin secretion in isolated islets. Insulin secretion in response to both subthreshold (2.8 mM) and suprathreshold (16.7 mM) glucose was augmented in islets from dexamethasone-treated rats (*n* = 10–12 wells, *p* < 0.05) and bezafibrate treatment did not affect these insulin responses (Figures 5(a) and 5(b)). Bezafibrate exerted a positive effect per se on islet response to 16.7 mM glucose. This effect is better described by the insulin responsiveness to high glucose as can be observed by the higher insulin release ratio between 16.7 mM and 2.8 mM glucose (Figure 5(c)). Thus, ex vivo insulin release was not downregulated in bezafibrate-treated rats.

## 4. Discussion

Elevation in plasma triacylglycerol/NEFA levels is commonly associated with a context of reduced insulin sensitivity [18, 19]. There is evidence that an increase in circulating NEFA is one of the causal factors of such peripheral insulin insensitivity [8, 20]. GC in excess exerts diabetogenic actions that include the increase in plasma triacylglycerol and NEFA levels and the reduction in peripheral insulin sensitivity [2–5, 16, 17], accompanied by an increase in blood glucose levels and the presence of glucose intolerance [2–7, 16, 17]. Here, we have provided the first demonstration that previous treatment with high-dose bezafibrate in adult male rats abolished the increase in plasma triacylglycerol levels caused by dexamethasone administration. The effect of bezafibrate on plasma triacylglycerol was associated with the prevention or attenuation of the increase in plasma NEFA and insulin, as well as blood glucose levels caused by GC administration. This normalization of plasma triacylglycerol in the BezaDexa group also paralleled the attenuation of glucose intolerance and prevention of any alterations in the peripheral insulin sensitivity caused by GC treatment.

Long-term treatment with bezafibrate exerted no impact on body mass gain and relative food intake, which were both reduced by the action of dexamethasone treatment (Figures 2(a)–2(e)). The reduction in body mass gain caused by GC results from reduced food intake [17, 21] and a negative hydric balance (increased water and sodium excretion without affecting water intake) [22]. This reduction in food intake is partially attributable to the anorexic effect of insulin and leptin on the hypothalamus, which is upregulated in dexamethasone-treated rats as they develop hyperinsulinemia and hyperleptinemia [3–5, 16, 17, 23]. Although the BezaDexa group had lower plasma insulin levels than in the Dexa group, it is feasible that these animals are more insulin sensitive (as our ITT experiments demonstrated), which favors the action of insulin in the hypothalamus (insulinemia in the BezaDexa group was between the Ctl and Dexa values).

Previous administration of bezafibrate was efficient in preventing any increase in plasma triacylglycerol levels caused by GC in the BezaDexa group, a result accompanied by a reproductive correction in plasma NEFA (especially in the fed state) and in blood glucose levels, with a milder effect on the insulinemia (Figures 3(a)–3(j)). This action of bezafibrate on the reduction of plasma triacylglycerol has been well documented in both rodents (rats and mice) and humans. The mechanisms by which bezafibrate acts include the increase in plasma triacylglycerol clearance through increased lipoprotein lipase and hepatic lipase activities [24, 25]. This increased plasmatic clearance of triacylglycerol is also associated with augmented mitochondrial performance and mass, energy expenditure, and a better metabolic flexibility [26, 27], which altogether support, at least in part, the disposal and oxidation of glucose and NEFA. The improvement in insulin sensitivity caused by bezafibrate administration, as discussed hereafter, may also explain the improvement of the insulin-suppressive effect on fat lipolysis and the hepatic gluconeogenesis (not directly investigated in the present study), favoring a better control of blood glucose and plasma lipids levels. There are numerous laboratory evidences demonstrating the positive impact of bezafibrate treatment on lipid and glucose metabolism in rodents, including diet-induced metabolic dysfunctions [28, 29] and monogenic/polygenic phenotypes of obesity/diabetes, such as db/db mice [30], TallyHo mice [27], OLEFT rats [13, 14], and models of diabetes induced by streptozotocin [26]. Studies with dyslipidemic nonobese humans [31] who are overweight [32] and have diabetes [33–35] also revealed the positive impact of bezafibrate treatment on lipid and glucose metabolism. Thus, bezafibrate may have a good spectrum of action and is well tolerated as a cotreatment [11]. Thus, we provided the first experimental evidence that high-dose bezafibrate can prevent the GC-induced elevation in triacylglycerol levels. However, these data are preliminary, and new studies should address reproducibility in animal and human studies, the efficacy of these drugs when administered at lower doses, together or after the introduction of GC, as well as the potential side effects not revealed in the present study.

One striking observation in the BezaDexa group was the fact that these animals did not present any alteration in the insulin sensitivity and had an improved glucose tolerance (Figures 4(a)–4(d)). There is evidence associating enhanced plasma NEFA values with reduction in peripheral insulin sensitivity [18, 19]. Among the free fatty acids (FFAs), palmitic acid is the most abundant in circulation and is a substrate for the synthesis of diacylglycerol (DAG) and ceramides, lipidic molecules that impair insulin signaling in peripheral tissues [8, 20]. Considering that rats submitted to the same protocol of GC treatment applied in the present study have decreased plasma levels of proinflammatory cytokines (i.e., tumor necrosis factor alpha, interleukin 1 beta) and normal plasma levels of interleukin 6 [36], we cannot attribute the insulin resistance in the Dexa group by an effect of such cytokines, which typically cause the downregulation of insulin signaling [37]. The reduction in insulin sensitivity in rats treated with dexamethasone includes decreased insulin signaling activation in the liver, skeletal muscle, and adipose tissue [3, 36, 38]. Thus, the activation of atypical and novel protein kinases C, which are lipid-mediated (i.e., by ceramides and DAG, respectively), may underlie this downregulation of insulin signaling [39]. There is evidence that bezafibrate treatment reduces the ectopic lipid content in skeletal muscle from rats fed with high fructose plus lard [28] and in the livers of normal rats [12]. Thus, we could consider that the prevention of lipids elevation in the circulation of the BezaDexa group might contribute to prevent a reduction in peripheral insulin sensitivity that could be related to the enhanced content of lipid in peripheral tissues, suggesting a contribution of excessive free fatty acids in the GC-induced insulin resistance.

The improvement in the glucose tolerance in the BezaDexa group may result from normal insulin sensitivity combined with enhanced islet insulin response to glucose (Figures 4(a)–4(e)). By the interpretation of the disposition index (the product of insulin sensitivity by the insulin secretion), we would not expect enhanced insulin secretion *in vivo* during the GTT experiment since these rats (BezaDexa) were not insulin-resistant. This benefit of bezafibrate on glucose tolerance is also reproducible in patients with DM2 after combined treatment of pioglitazone and bezafibrate [40]. In this study, authors observed that this attenuation of glucose tolerance is due to a reduction in postmeal-induced insulin secretion rather than reduction in glucagon secretion, rendering a diminished plasma insulin/glucagon ratio. Similar results on insulin/glucagon ratio, due to reduced insulin secretion rather than increase glucagon secretion, after stimulation with arginine, were observed in health subjects [41] and hypertriglyceridemic patients [42] treated with 2 grams of clofibrate for 7 and 2–4 months, respectively. These findings support our results in the BezaDexa rats that had decreased insulinemia (Figures 3(e) and 3(j)) and insulin secretion during the ipGTT (Figure 4(c)). However, there is evidence for a direct effect of bezafibrate on glucose-stimulated insulin secretion in islets isolated from rats [43], consistent with the results of insulin secretion in response to 16.7 mM glucose in islets isolated from the Beza and BezaDexa groups (Figures 5(b) and 5(c)). Notably, the highest plasma insulin levels observed in the Dexa group (under fasting and fed conditions) involved not only the endocrine pancreas adherence to the disposition index (that demand increased islet function) but also a reduction in plasma insulin clearance. A previous study involving rats treated with the same dexamethasone protocol demonstrated that the insulin-degrading enzyme activity in the liver is reduced in GC-treated rats, explaining, in part, the hyperinsulinemia observed in these animals [44]. Nonetheless, we cannot rule out the direct action of GCs on gene transactivation of gluconeogenic enzymes (i.e., phosphoenolpyruvate carboxykinase and glucose-6-phosphatase) that results in augmented hepatic glucose output when upregulated by dexamethasone treatment [36, 45], an action that likely outweighs the bezafibrate benefit.

## 5. Conclusions

In conclusion, prevention of the elevation of plasma triacylglycerol levels through high-dose bezafibrate treatment abolishes the insulin resistance and attenuates the glucose intolerance caused by dexamethasone administration in rats. These observations open the possibility of a combination of lipid-lowering drugs with GCs therapies in clinical practice, aiming to reduce the most prevalent adverse effects of GC on metabolism, insulin resistance, and glucose intolerance [46].

## Figures and Tables

**Table 1:** The relative organ and tissue masses and hepatic glycogen data at the day of euthanasia.

	Ctl	Dexa	Beza	BezaDexa
^1^Retroperitoneal fat	1.1 ± 0.3	1.1 ± 0.4	1.0 ± 0.4	1.1 ± 0.4
^1^Epididymal fat	1.1 ± 0.2	1.3 ± 0.3	1.1 ± 0.3	1.2 ± 0.3
^1^Visceral fat (both)	2.2 ± 0.4	2.4 ± 0.7	2.1 ± 0.7	2.3 ± 0.6
^2^Spleen	193 ± 17	124 ± 9^∗^	200 ± 22	133 ± 16^∗^
^3^Hepatic glycogen content	0.9 ± 0.5	4.6 ± 1.1^∗^	0.6 ± 0.2	0.7 ± 0.4^#^

^1^(g/100 g, b.m.); ^2^(mg/100 g, b.m.); ^3^(mg/g tissue), read methods for details. Results are expressed as mean ± SD. ^∗^Significantly different vs. the respective control group (dexamethasone effect) and ^#^significantly different vs. the respective control group (bezafibrate effect) using two-way ANOVA with Tukey's post hoc test. *n* = 7–8, *p* < 0.05. 1 and 2 are equal to (g/b.m) × 100 or (mg/b.m.) × 100, respectively.

## Data Availability

The datasets generated to support the findings of this study are available from the corresponding author upon reasonable request.

## References

[1] Nunes E. A., Rafacho A. (2017). Implications of palmitoleic acid (palmitoleate) on glucose homeostasis, insulin resistance and diabetes. *Current Drug Targets*.

[2] Pasieka A., Rafacho A. (2016). Impact of glucocorticoid excess on glucose tolerance: clinical and preclinical evidence. *Metabolites*.

[3] Burén J., Lai Y. C., Lundgren M., Eriksson J. W., Jensen J. (2008). Insulin action and signalling in fat and muscle from dexamethasone-treated rats. *Archives of Biochemistry and Biophysics*.

[4] Angelini N., Rafacho A., Boschero A. C., Bosqueiro J. R. (2010). Involvement of the cholinergic pathway in glucocorticoid-induced hyperinsulinemia in rats. *Diabetes Research and Clinical Practice*.

[5] Rafacho A., Abrantes J. L. F., Ribeiro D. L. (2011). Morphofunctional alterations in endocrine pancreas of short- and long-term dexamethasone-treated rats. *Hormone and Metabolic Research*.

[6] Schneiter P., Tappy L. (1998). Kinetics of dexamethasone-induced alterations of glucose metabolism in healthy humans. *American Journal of Physiology-Endocrinology and Metabolism*.

[7] Ahrén B. (2008). Evidence that autonomic mechanisms contribute to the adaptive increase in insulin secretion during dexamethasone-induced insulin resistance in humans. *Diabetologia*.

[8] Hage Hassan R., Bourron O., Hajduch E. (2014). Defect of insulin signal in peripheral tissues: important role of ceramide. *World Journal of Diabetes*.

[9] Nayak I. M. N., Narendar K., PA M., Jamadar M. G., Kumar V. H. (2017). Comparison of pioglitazone and metformin efficacy against glucocorticoid induced atherosclerosis and hepatic steatosis in insulin resistant rats. *Journal of Clinical and Diagnostic Research*.

[10] Seelig E., Meyer S., Timper K. (2017). Metformin prevents metabolic side effects during systemic glucocorticoid treatment. *European Journal of Endocrinology*.

[11] Goldenberg I., Benderly M., Goldbourt U. (2008). Update on the use of fibrates: focus on bezafibrate. *Vascular Health and Risk Management*.

[12] Karahashi M., Hoshina M., Yamazaki T. (2013). Fibrates reduce triacylglycerol content by upregulating adipose triglyceride lipase in the liver of rats. *Journal of Pharmacological Sciences*.

[13] Jia D., Yamamoto M., Otani M., Otsuki M. (2004). Bezafibrate on lipids and glucose metabolism in obese diabetic Otsuka Long-Evans Tokushima fatty rats. *Metabolism*.

[14] Mori Y., Oana F., Matsuzawa A., Akahane S., Tajima N. (2004). Short-term effect of bezafibrate on the expression of adiponectin mRNA in the adipose tissues: a study in spontaneously type 2 diabetic rats with visceral obesity. *Endocrine*.

[15] Pispirigos K., Simopoulos K., Kouskoukis K., Kounis N., Avramopoulos A. (1999). Evaluation of kidney and liver subacute toxicity induced by bezalip - pravastatin. Lopid antihyperlipidaemic compounds in rats. *IUBMB Life*.

[16] Giozzet V. A. G., Rafacho A., Boschero A. C., Carneiro E. M., Bosqueiro J. R. (2008). Dexamethasone treatment in vivo counteracts the functional pancreatic islet alterations caused by malnourishment in rats. *Metabolism*.

[17] dos Santos C., Ferreira F. B. D., Gonçalves-Neto L. M., Taboga S. R., Boschero A. C., Rafacho A. (2014). Age- and gender-related changes in glucose homeostasis in glucocorticoid-treated rats. *Canadian Journal of Physiology and Pharmacology*.

[18] Reaven G. M., Hollenbeck C., Jeng C. Y., Wu M. S., Chen Y. D. I. (1988). Measurement of plasma glucose, free fatty acid, lactate, and insulin for 24 h in patients with NIDDM. *Diabetes*.

[19] Boden G. (1997). Role of fatty acids in the pathogenesis of insulin resistance and NIDDM. *Diabetes*.

[20] Shulman G. I. (2000). Cellular mechanisms of insulin resistance. *The Journal of Clinical Investigation*.

[21] Gonçalves-Neto L. M., Ferreira F. B., Souza L. (2014). Disruption of glucose tolerance caused by glucocorticoid excess in rats is partially prevented, but not attenuated, by arjunolic acid. *Indian Journal of Experimental Biology*.

[22] Thunhorst R. L., Beltz T. G., Johnson A. K. (2007). Glucocorticoids increase salt appetite by promoting water and sodium excretion. *American Journal of Physiology-Regulatory, Integrative and Comparative Physiology*.

[23] Caldefie-Ch[eacute]zet F., Moinard C., Minet-Quinard R., Gachon F., Cynober L., Vasson M. P. (2001). Dexamethasone treatment induces long-lasting hyperleptinemia and anorexia in old rats. *Metabolism*.

[24] Miller D. B., Spence J. D. (1998). Clinical pharmacokinetics of fibric acid derivatives (fibrates). *Clinical Pharmacokinetics*.

[25] Kazumi T., Yoshino G., Iwai M. (1990). Long-term effects of bezafibrate on in vivo VLDL-triglyceride production in the rat. *Diabetes Research and Clinical Practice*.

[26] Franko A., Huypens P., Neschen S. (2016). Bezafibrate improves insulin sensitivity and metabolic flexibility in STZ-induced diabetic mice. *Diabetes*.

[27] Franko A., Neschen S., Rozman J. (2017). Bezafibrate ameliorates diabetes via reduced steatosis and improved hepatic insulin sensitivity in diabetic TallyHo mice. *Molecular Metabolism*.

[28] Matsui H., Okumura K., Kawakami K., Hibino M., Toki Y., Ito T. (1997). Improved insulin sensitivity by bezafibrate in rats: relationship to fatty acid composition of skeletal-muscle triglycerides. *Diabetes*.

[29] Si X., Webb R. C., Richey J. M. (1999). Bezafibrate, an anti-hypertriglyceridemic drug, attenuates vascular hyperresponsiveness and elevated blood pressure in fructose-induced hypertensive rats. *Canadian Journal of Physiology and Pharmacology*.

[30] Yajima K., Hirose H., Fujita H. (2003). Combination therapy with PPAR*γ* and PPAR*α* agonists increases glucose-stimulated insulin secretion in *db*/*db*mice. *American Journal of Physiology-Endocrinology and Metabolism*.

[31] Inoue I., Takahashi K., Katayama S. (1994). Improvement of glucose tolerance by bezafibrate in non-obese patients with hyperlipidemia and impaired glucose tolerance. *Diabetes Research and Clinical Practice*.

[32] Karhapää P., Uusitupa M., Voutilainen E., Laakso M. (1992). Effects of bezafibrate on insulin sensitivity and glucose tolerance in subjects with combined hyperlipidemia. *Clinical Pharmacology and Therapeutics*.

[33] Riccardi G., Genovese S., Saldalamacchia G. (1989). Effects of bezafibrate on insulin secretion and peripheral insulin sensitivity in hyperlipidemic patients with and without diabetes. *Atherosclerosis*.

[34] Jones I. R., Swai A., Taylor R., Miller M., Laker M. F., Alberti K. G. M. (1990). Lowering of plasma glucose concentrations with bezafibrate in patients with moderately controlled NIDDM. *Diabetes Care*.

[35] Taniguchi A., Fukushima M., Sakai M. (2001). Effects of bezafibrate on insulin sensitivity and insulin secretion in non-obese Japanese type 2 diabetic patients. *Metabolism*.

[36] Motta K., Barbosa A. M., Bobinski F., Boschero A. C., Rafacho A. (2015). JNK and IKK*β* phosphorylation is reduced by glucocorticoids in adipose tissue from insulin-resistant rats. *The Journal of Steroid Biochemistry and Molecular Biology*.

[37] Khodabandehloo H., Gorgani-Firuzjaee S., Panahi G., Meshkani R. (2016). Molecular and cellular mechanisms linking inflammation to insulin resistance and *β*-cell dysfunction. *Translational Research*.

[38] Saad M. J., Folli F., Kahn J. A., Kahn C. R. (1993). Modulation of insulin receptor, insulin receptor substrate-1, and phosphatidylinositol 3-kinase in liver and muscle of dexamethasone-treated rats. *The Journal of Clinical Investigation*.

[39] Rafacho A., Nunes E. A., Bordin S. (2015). How much we know about the attenuation of insulin signaling in the adipose tissue caused by glucocorticoid treatment?. *Inflammation and Cell Signaling*.

[40] Balasubramanian R., Gerrard J., Dalla Man C. (2010). Combination peroxisome proliferator-activated receptor *γ* and *α* agonist treatment in type 2 diabetes prevents the beneficial pioglitazone effect on liver fat content. *Diabetic Medicine*.

[41] Eaton R. P., Schade D. S. (1974). Effect of clofibrate on arginine-stimulated glucagon and insulin secretion in man. *Metabolism*.

[42] Tiengo A., Muggeo M., Assan R., Fedele D., Crepaldi G. (1975). Glucagon secretion in primary endogenous hypertriglyceridemia before and after clofibrate treatment. *Metabolism*.

[43] Yoshikawa H., Tajiri Y., Sako Y., Hashimoto T., Umeda F., Nawata H. (2001). Effects of bezafibrate on *β*-cell function of rat pancreatic islets. *European Journal of Pharmacology*.

[44] Protzek A. O. P., Rezende L. F., Costa-Júnior J. M. (2016). Hyperinsulinemia caused by dexamethasone treatment is associated with reduced insulin clearance and lower hepatic activity of insulin-degrading enzyme. *The Journal of Steroid Biochemistry and Molecular Biology*.

[45] Wang X. L., Herzog B., Waltner-Law M., Hall R. K., Shiota M., Granner D. K. (2004). The synergistic effect of dexamethasone and all-*trans*-retinoic acid on hepatic phosphoenolpyruvate carboxykinase gene expression involves the coactivator p300. *Journal of Biological Chemistry*.

[46] Destro M. (2011). *Avaliação da participação dos ácidos graxos nas adaptações das ilhotas pancreáticas à resistência periférica à insulina pelo tratamento com dexametasona*.

